# Ezetimibe/simvastatin vs simvastatin in coronary heart disease patients with or without diabetes

**DOI:** 10.1186/1476-511X-9-80

**Published:** 2010-07-27

**Authors:** Carlo M Rotella, Augusto Zaninelli, Cristina Le Grazie, Mary E Hanson, Gian Franco Gensini

**Affiliations:** 1 Dipartimenti Fisiopatologia Clinica, Universitàd i Firenze, Viale Pieraccini 6, 50139, Florence, Italy; 2 Department of Internal Medicine and Cardiology, University of Florence, Florence, Italy; 3 Medical and Scientific Affairs, Merck Sharpe and Dohme, Milano, Italy; 4 Global Scientific & Medical Publications, Merck, North Wales, PA, USA

## Abstract

**Background:**

Treatment guidelines recommend LDL-C as the primary target of therapy in patients with hypercholesterolemia. Moreover, combination therapies with lipid-lowering drugs that have different mechanisms of action are recommended when it is not possible to attain LDL-C targets with statin monotherapy. Understanding which treatment or patient-related factors are associated with attaining a target may be clinically relevant.

**Methods:**

Data were pooled from two multicenter, randomized, double-blind studies. After stabilization on simvastatin 20 mg, patients with coronary heart disease (CHD) alone and/or type 2 diabetes mellitus (T2DM) were randomized to ezetimibe 10 mg/simvastatin 20 mg (EZ/Simva) or simvastatin 40 mg. The change from baseline in low-density lipoprotein cholesterol (LDL-C), total cholesterol (TC), high-density lipoprotein cholesterol (HDL-C), TC/HDL-C ratio, triglycerides, and the proportion of patients achieving LDL-C < 2.6 mmol/L (100 mg/dL) after 6 weeks of treatment were assessed, and factors significantly correlated with the probability of achieving LDL-C < 2.6 mmol/L in a population of high cardiovascular risk Italian patients were identified. A stepwise logistic regression model was conducted with LDL-C < 2.6 mmol/L at endpoint as the dependent variable and study, treatment, gender, age (≥65 years or < 65 years), as independent variables and baseline LDL-C (both as continuous and discrete variable).

**Results:**

EZ/Simva treatment (N = 93) resulted in significantly greater reductions in LDL-C, TC, and TC/HDL-C ratio and higher attainment of LDL-C < 2.6 mmol/L vs doubling the simvastatin dose to 40 mg (N = 106). Study [including diabetic patients (OR = 2.9, p = 0.003)], EZ/Simva treatment (OR = 6.1, p < 0.001), and lower baseline LDL-C (OR = 0.9, p = 0.001) were significant positive predictors of LDL-C target achievement. When baseline LDL-C was expressed as a discrete variable, the odds of achieving LDL-C < 2.6 mmol/L was 4.8 in favor of EZ/Simva compared with Simva 40 mg (p < 0.001), regardless of baseline LDL-C level.

**Conclusion:**

EZ/Simva is an effective therapeutic option for patients who have not achieved recommended LDL-C treatment targets with simvastatin 20 mg monotherapy.

**Trial Registration:**

Clinical trial registration numbers: NCT00423488 and NCT00423579

## Introduction

Treatment guidelines recommend reducing low-density lipoprotein cholesterol (LDL-C) as the primary target of therapy for patients with hypercholesterolemia [1-4]. Although therapeutic lifestyle changes (smoking cessation, increased activity, and reduced fat intake) are the cornerstone of population-based interventions, often they are not sufficient to achieve recommended treatment targets. HMG-CoA reductase inhibitors (statins) are the first line of lipid-modifying treatment, the benefits of which have been shown across coronary heart disease (CHD) risk strata. These benefits have also been documented in both genders, across a range of age groups, and in patients with or without diabetes or hypertension [5]. European, Canadian and US treatment guidelines recommend combination therapy with lipid-lowering drugs that have different mechanisms of action when it is not possible to attain the lowest LDL-C targets with statin monotherapy [1,3,4].

Ezetimibe is a cholesterol absorption inhibitor that blocks biliary and dietary cholesterol absorption at the brush border of the intestine without affecting absorption of fat-soluble vitamins and triglycerides [6]. In patients with primary hypercholesterolemia, studies of ezetimibe monotherapy have demonstrated significantly greater reductions in LDL-C levels compared with placebo [7,8], and when coadministered with a statin, ezetimibe has been shown to be significantly more effective at reducing LDL-C levels compared with statin monotherapy (reviewed in [9]). These results appear to be consistent across risk strata [10-12] and age groups [13] and in patients with metabolic syndrome [14] or type 2 diabetes mellitus (T2DM) [15,16].

When confronted with the choice of an alternative treatment in high-risk patients not at target with statin monotherapy, it would be clinically relevant to know which treatment or patient-related factors might correlate with higher odds of attaining a target. The aim of this pooled analysis of data from two multicenter, randomized, double-blind studies in high-risk patients not at target with simvastatin 20 mg/day [17,18] was to evaluate the factors significantly correlated with the probability of achieving the National Cholesterol Education Program Adult Treatment Panel III (NCEP ATP III) recommended LDL-C target < 2.6 mmol/L (100 mg/dL) after 6 weeks of treatment with EZ/Simva 10/20 mg vs doubling the dose of simvastatin to 40 mg. The combined analysis of data from these two studies with the same design and treatment regimens provides a larger population of patients at high cardiovascular risk in which to evaluate the efficacy of the two treatment regimens.

## Methods

### Study design

The design was similar for both studies. Briefly, both were multicenter, randomized, parallel-groups, double-blind, double-dummy, placebo-controlled studies. Both protocols were reviewed and approved by an Independent Ethics Committee at each participating center; and patients provided written, informed consent prior to any study-related procedure being started. The studies were conducted under the provisions of the Declaration of Helsinki and in accordance with the International Conference on Harmonization Consolidated Guidelines on Good Clinical Practice.

### Study population

In the LEAD study only [17], patients were required to have adequately controlled T2DM (defined as fasting plasma glucose > 126 mg/dL and hemoglobin (Hb) A_1c _≤ 9.0%) of at least 12 months duration. In both studies (LEAD and DIALOGUE), men and women ≥18 years and ≤ 75 years of age with documented CHD, including stable angina with evidence of ischemia on exercise testing; history of myocardial infarction, percutaneous transluminal coronary intervention, atherothrombotic cerebrovascular disease, unstable angina or non-Q wave myocardial infarction; or symptomatic peripheral vascular disease, who were taking a stable daily dose of simvastatin 20 mg for 6 weeks with good compliance (80% of daily doses for the 6 weeks prior to baseline visit), and had LDL-C concentration ≥ 2.6 mmol/L (100 mg/dL) to ≤ 4.1 mmol/L (160 mg/dL) were eligible for randomization. Patients were instructed to follow a healthy lifestyle (cholesterol-lowering diet and exercise) throughout the study. In addition, subjects were required to have triglyceride concentrations < 3.99 mmol/L (350 mg/dL), liver transaminases [alanine aminotransferase (ALT) or aspartate aminotransferase (AST)] and creatine phosphokinase (CK) < 50% above the upper limit of normal (ULN) with no active liver disease, and hematology, blood chemistry, and urinalysis within normal limits. Women of childbearing potential were required to use birth control considered effective by the investigators [17,18].

Patients were excluded if they had Class III or IV congestive heart failure, uncontrolled cardiac arrhythmia; recent (within 3 months of randomization) myocardial infarction, acute coronary insufficiency, coronary artery bypass surgery, or angioplasty; unstable or severe peripheral artery disease; newly diagnosed or unstable angina pectoris, uncontrolled hypertension (treated or untreated); uncontrolled endocrine or metabolic disease known to influence serum lipids or lipoproteins; impaired renal function (creatinine > 2.0 mg/dL) or nephrotic syndrome; or were taking any lipid-lowering agents, fibrates, resins or niacin, or prescription and/or over-the-counter-drugs with the potential for significant lipid effects (other than study drug) or with potential drug interactions with the statins.

### Efficacy measures

Efficacy endpoints were those that were prespecified in the trials prior to pooling the data. The percent change in LDL-C from baseline to endpoint after 6 weeks of treatment, the percentage of patients who reached LDL-C < 2.6 mmol/L (100 mg/dL) at endpoint, and the percent change from baseline to endpoint in total cholesterol, high-density lipoprotein cholesterol (HDL-C), total cholesterol/HDL-C and triglycerides were assessed after 6 weeks of treatment. LDL-C measurements were calculated by the Friedewald equation (all patients included in the study had triglycerides < 4.52 mmol/L [ < 400 mg/dL]). Study, treatment, gender, age, and baseline LDL-C were assessed as potential predictors of LDL-C target achievement.

### Tolerability

Adverse events were summarized by system organ class and specific adverse experience term. Laboratory tests included complete blood count, total protein, albumin, calcium, inorganic phosphorus, fasting plasma glucose (FPG), blood urea nitrogen (BUN), uric acid, total bilirubin, alkaline phosphatase, ALT, AST, gamma glutamyl transpeptidase (GGT), serum creatinine, thyroid stimulating hormone (TSH; baseline only), HbA_1c_, sodium, potassium, chloride, CK; and urinalysis.

### Statistics

Efficacy endpoints were assessed in the intent-to-treat (ITT) population, which included all subjects who were randomized, had taken at least one dose of study drug, and had at least one measurement at baseline and after the start of treatment, using the ANOVA model, which included terms for treatment effect. Factors significantly correlated with the probability of achieving LDL-C < 2.6 mmol/L were assessed using a stepwise logistic regression analysis with LDL-C < 2.6 mmol/L at endpoint as the dependent variable (yes/no). Independent variables were study (T2DM, yes or no), treatment, gender, age (≥65 years or < 65 years), and baseline LDL-C, which was both a continuous and discrete variable. The safety population included all randomized patients who took at least one dose of study drug. The incidence of adverse events was compared between treatments using the Fisher exact test with Yates correction if applicable.

## Results

### Population

The ITT population included 93 patients treated with EZ/Simva 10/20 mg (n = 37 from LEAD and n = 56 from DIALOGUE) and 106 patients treated with Simva 40 mg (n = 50 from LEAD and n = 56 from DIALOGUE). Baseline characteristics for the combined population are shown in Table 1. The mean age (± standard deviation) was 63 ± 8 years in the EZ/Simva 10/20 mg group and 63 ± 7 years in the Simva 40 mg group. All patients were Caucasian and most patients were male (55% in the EZ/Simva 10/20 mg group and 66% in the Simva 40 group). Both treatment groups were generally well-matched for demographic data, cardiovascular risk factors, and baseline laboratory values. The mean LDL-C was 3.3 mmol/L (126.2 mg/dL) in the EZ/Simva 10/20 mg group and 3.3 mmol/L (127.1 mg/dL) in the Simva 40 mg group. Ischemic heart disease was the most common form of CHD reported, with 40 (43%) patients in the EZ/Simva 10/20 mg group and 49 (46%) patients in the Simva 40 mg group (Table 1). Major differences between the studies included the level of fasting plasma glucose, which was higher in the diabetic patients compared with the non-diabetic patients, and more women in the non-diabetic population vs the diabetic population [17,18].

**Table 1:** Baseline patient characteristics

Demographics	EZ/Simva 10/20 (N = 93)	Simva 40 (N = 106)
Age, yrs mean (SD)	62.8 (7.9)	62.9 (7.1)

Females	42 (45%)	36 (34%)

Body mass index, kg/m2, mean (SD)	73.7 (11.6)	75.1 (12.7)

Current smokers	16 (17.2%)	21 (19.8%)

Hypertension	58 (62.4%)	56 (52.8%)

Diabetes	37 (39.8%)	50 (47.2%)

**Laboratory values** **mean (SD)**		

LDL-C (mmol/L)	3.27 (0.47)	3.29 (0.44)

TC (mmol/L)	5.18 (0.58)	5.18 (0.56)

HDL-C (mmol/L)	1.26 (0.30)	1.19 (0.26)

TC/HDL-C	4.31 (1.03)	4.54 (1.00)

Triglycerides (mmol/L)	1.45 (0.62)	1.52 (0.59)

AST (U/L)	19.8 (5.3)	20.6 (4.7)

ALT (U/L)	22.7 (9.5)	25.5 (9.7)

CK (U/L)	97.0 (44.1)	110.7 (46.2)

**Prevalence of cardiovascular diseases, n (%)**		

Cerebrovascular disease	30 (32.3%)	30 (28.3%)

Peripheral vascular disease	32 (34.4%)	39 (36.8%)

Ischemic heart disease	40 (43.0%)	49 (46.2%)

ALT = alanine aminotransferase; AST = aspartate aminotransferase; CK = creatine kinase;

EZ = ezetimibe; HDL-C = high-density lipoprotein cholesterol; LDL-C = low-density lipoprotein cholesterol; Simva = simvastatin; TC = total cholesterol

### Efficacy

**Figure 1:**
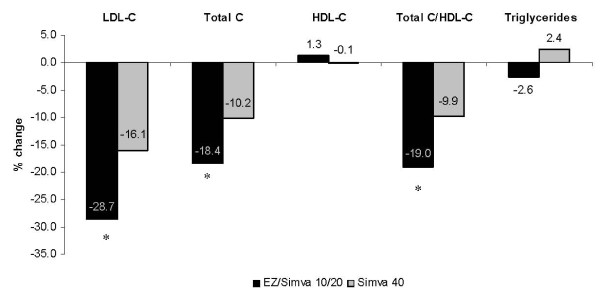
**Change from treated baseline in LDL-C and other lipids after 6 weeks of treatment**. *p < 0.01

**Figure 2:**
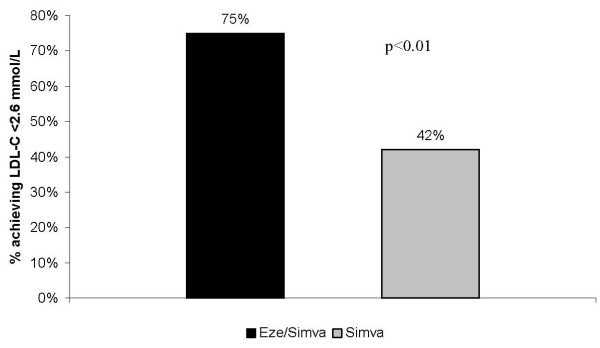
**Proportion of patients achieving LDL-C < 2.6 mmol/L (100 mg/dL) after 6 weeks of treatment with EZ/Simva 10/20 mg or Simva 40 mg**. p < 0.01

Compared with doubling the dose of simvastatin to 40 mg, treatment with EZ/Simva 10/20 mg resulted in significantly greater reductions from baseline in LDL-C, total cholesterol, and total cholesterol/HDL-C ratio (Figure 1; all p < 0.01); and significantly more patients treated with EZ/Simva 10/20 mg achieved LDL-C < 2.6 mmol/L after 6 weeks of treatment (Figure 2; p < 0.01). Changes in HDL-C and triglycerides were similar between treatment groups (Figure 1).

**Figure 3:**
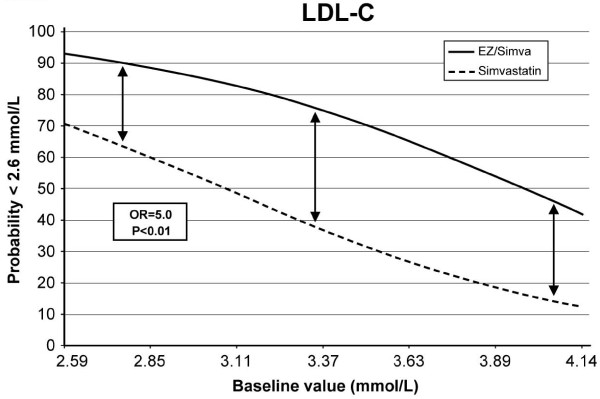
**The odds of achieving LDL-C < 2.6 mmol/L after 6 weeks of treatment with EZ/Simva 10/20 mg or Simva 40 mg by baseline LDL-C level = 5.0**. p < 0.01

Among the independent variables, participation in the LEAD study (which included patients with T2DM; odds ratio [OR] = 2.9, 95% confidence interval [CI]: 1.4-5.9; p = 0.003) and EZ/Simva 10/20 treatment (OR = 6.1, 95% CI: 2.9-12.4; p < 0.001) were significant, positive independent predictors, and higher baseline LDL-C was a significant, negative independent predictor (OR = 0.9, 95% CI: 0.93-0.97; p = 0.001) of achieving LDL-C < 2.6 mmol/L. When study was removed from the model, EZ/Simva treatment remained a significant positive predictor (OR = 5.0, 95% CI: 2.6-9.9; p < 0.01; Figure 3), and higher baseline LDL-C remained a significant negative predictor (OR = 0.9; 95% CI: 0.93-0.97; p < 0.01) of LDL-C target achievement.

Tolerability results are summarized in Table 2. There was no significant difference in the proportion of patients who reported adverse events between treatment groups (p = 0.606). No significant differences between groups were observed in the number and rate of drug-related adverse events, which were reported in 9.8% of patients in the EZ/Simva 10/20 mg group and in 6.3% of patients in the Simva 40 mg group (p = 0.500). There were few discontinuations due to treatment-related adverse events (only 1 patient in the EZ/Simva 10/20 mg group and 2 in the Simva 40 mg group). Two serious adverse events were reported: one in the EZ/Simva 10/20 mg group (bone fracture) and one in the Simva 40 mg group (transient ischemic attack). Neither was considered drug-related. There were no reports of increased ALT or AST ≥ 3 × ULN or CK ≥ 5-10 × ULN, and no deaths occurred at any time during either study in either treatment group.

**Table 2:** Summary of tolerability data

	EZ/Simva 10/20 mg	Simva 40 mg	p-value
**Safety population **n (%)	N = 102	N = 111	

With adverse events	18 (17.6)	23 (20.7)	0.606

With treatment-related adverse events	10 (9.8)	7 (6.3)	0.500

Discontinued due to treatment-related adverse events	1 (1.0)	2 (1.8)	0.999

Serious adverse events	1 (0.1)*	1 (0.1)^†^	1.000

ALT/AST ≥ 3 × ULN	0	0	--

CK ≥ 5-10 × ULN	0	0	--

*Bone fracture non-drug related

**^†^**Transient ischemic attack, non-drug related

ULN = upper limit of normal

## Discussion

The results of this pooled analysis were consistent with those of the individual studies, confirming that, compared with doubling the dose of simvastatin to 40 mg, treatment with EZ/Simva 10/20 mg produced significantly greater reductions in LDL-C, non-HDL-C, and total cholesterol/HDL-C ratio; and significantly higher proportions of patients achieved the NCEP ATP III-recommended treatment target of LDL-C < 2.6 mmol/L [17,18]. In addition, participation in the LEAD study (which included CHD patients with T2DM), lower baseline LDL-C, and treatment with EZ/Simva 10/20 mg (regardless of baseline LDL-C) were positive predictors of achieving LDL-C < 2.6 mmol/L in this high cardiovascular (CV) risk population with established CHD.

The results of this pooled analysis are in agreement with those of previous studies that assessed the efficacy of ezetimibe coadministered with a statin in high CV risk populations pre-treated with statins but not at LDL-C goal[10,12,19-21]. Taken together, these studies demonstrate that patients with hypercholesterolemia and CHD, who are at high CV risk and have not attained recommended LDL-C treatment goals while on statin, may benefit from ezetimibe added to statin therapy through significantly greater improvements in LDL-C and higher attainment of LDL-C treatment targets.

Knowledge of the factors that predict successful treatment may have implications in the management of high CV risk patients with hypercholesterolemia. It has been shown that higher baseline LDL-C was a significant negative predictor of LDL-C goal attainment, and this is consistent with the results reported here [22,23]. Other analyses have reported that two important independent predictors of LDL-C goal attainment were appropriate drug therapy and statin compliance [24,25]. Not surprisingly, exercise, dietary compliance, and weight loss were also significant predictors of goal attainment [24]. Although compliance and therapeutic lifestyle changes were not assessed in this study, the comparison of the two treatment regimens did indicate that treatment with the combination of EZ/Simva 10/20 mg was a significant positive predictor of achieving LDL-C < 2.6 mmol/L. Moreover, baseline LDL-C level did not impact this positive treatment effect, indicating that the odds in favor of the combination EZ/Simva are maintained for baseline LDL-C values within the range observed in this population. These results indicate that EZ/Simva, by targeting both the hepatic synthesis and the intestinal absorption of cholesterol, may be an effective therapeutic option for patients who have not achieved recommended LDL-C treatment targets with statin monotherapy.

Several studies or post hoc analyses have assessed LDL-C lowering and achievement of recommended treatment targets in patients with T2DM treated with different doses of EZ/Simva vs statin monotherapy and showed that the combination provided consistently greater lipid-lowering and higher goal attainment than statin monotherapy in patients with and without T2DM [14,16,26,27]. Most analyses did not include a comparison of goal attainment between patient groups, nor an analysis of factors that predict the odds of achieving goal. One report, however, showed that more patients in the diabetes group achieved the recommended LDL-C goal compared with their non-diabetes counterparts (83.6% versus 67.2%), although this result was not statistically significant after adjusting for differences in baseline LDL-C levels [28]. A preliminary report of a post hoc analysis of patients in the IN-CROSS study demonstrated a significant interaction for LDL-C lowering, indicating larger between-group reductions in patients with T2DM versus those without T2DM [29]. The results of the logistic regression analysis presented here indicated nearly three-fold greater odds of achieving the recommended LDL-C target of < 2.6 mmol/L for patients included in the LEAD study, which included only CHD patients with T2DM, vs those included in the DIALOGUE study, which included only CHD patients without T2DM. Taken together, these results suggest that baseline LDL-C may not be the only factor in achievement of LDL-C < 2.6 mmol/L for patients with T2DM when compared with patients without T2DM, since baseline levels were nearly identical in both studies (~3.3 mmol/L).

Poor response to statin monotherapy and suboptimal goal achievement in clinical practice may be due to a number of reasons [30]. Inter-individual variability in LDL-C lowering has been reported with both high-potency statins [31] and ezetimibe [32]; and a negative correlation between the response to statins and the subsequent response to ezetimibe has been shown in patients with genotype-confirmed heterozygous familial hypercholesterolemia [32]. The role of cholesterol homeostasis in response to statins is complex and unclear, and studies aiming to identify the serum markers that may be involved in the putative pathway have not yielded conclusive results, especially in patients with diabetes [33-38]. From a clinical perspective, however, it is evident that there is no easily accessible test to determine if a diabetic patient behaves as a "high absorber" or as a "high synthesizer" of cholesterol, allowing clinicians the ability to target lipid-lowering treatment accordingly. The response to statin monotherapy with appropriate potency, dose, and duration is likely the only way to assess if a patient will attain treatment targets with statin monotherapy. The results of clinical trials support a complementary approach that targets the synthesis and the absorption of cholesterol to improve the lipid profile of patients who show a poor response to statin monotherapy [11,12,20].

The tolerability profiles of both treatment regimens were similar. Although pooling the data from two studies increased the power of the statistical analysis, the treatment group sizes were relatively small, and the studies were not of sufficient duration to detect the presence of very rare adverse events. Despite these limitations, the tolerability results are consistent with expectations for these drugs at the doses given and with previous trials in high-risk CV patients with and without T2DM [14-16].

This pooled analysis supports and extends previous findings that demonstrate the significantly greater efficacy of the combination EZ/Simva compared with statin monotherapy in LDL-C reductions and NCEP-recommended target achievement in high CV risk patients with and without T2DM. In particular, patients had significantly higher odds of achieving the NCEP-recommended LDL-C target when treated with combination therapy compared with doubling the simvastatin dose, even at low baseline LDL-C levels. In addition, EZ/Simva combination therapy was a well-tolerated treatment. In high CV risk patients with hypercholesterolemia who have not achieved individual treatment targets on statin monotherapy, combination therapies such as EZ/Simva may provide a superior therapeutic option for improving their lipid profiles. This analysis did not assess clinical outcomes; however, trials are ongoing to measure the efficacy of EZ/Simva on clinical outcomes.

## List of Abbreviations

ALT: alanine aminotransferase; AST: aspartate aminotransferase; CHD: coronary heart disease; CK: creatine kinase; CV: cardiovascular; EZ/Simva: ezetimibe 10 mg/simvastatin 20 mg; HDL-C: high-density lipoprotein cholesterol; ITT: intent-to-treat; LDL-C: low-density lipoprotein cholesterol; NCEP-ATP II: National Cholesterol Education Program Adult Treatment Panel III; TC: total cholesterol; T2DM: type 2 diabetes mellitus; ULN: upper limit of normal

## Competing interests

CMR, AZ, and GFG report having no conflicts of interest to declare regarding this manuscript. CLG was an employee of Merck Sharp & Dohme at the time this study was conducted and the manuscript was written; MEH is an employee of Merck

## Authors' contributions

CMR and AZ conceived, designed or planned the study; collected or assembled the data; performed or supervised analyses; and interpreted the results; provided substantive suggestions for revision or critically reviewed the manuscript; and provided study materials or patients and statistical expertise. CLG conceived, designed or planned the study; wrote sections of the initial draft, performed or supervised analyses; and interpreted the results; provided substantive suggestions for revision or critically reviewed the manuscript; and provided administrative, technical or logistic support. MEH interpreted the results; wrote sections of the initial draft; and provided administrative, technical or logistic support. GFG conceived, designed or planned the study; collected or assembled the data; performed or supervised analyses; and interpreted the results; provided substantive suggestions for revision or critically reviewed the manuscript; and provided study materials or patients. All authors reviewed and approved the final version of the paper and approved of its submission to *Lipids in Health and Disease*.
